# Runoff prediction of lower Yellow River based on CEEMDAN–LSSVM–GM(1,1) model

**DOI:** 10.1038/s41598-023-28662-5

**Published:** 2023-01-27

**Authors:** Shaolei Guo, Yihao Wen, Xianqi Zhang, Haiyang Chen

**Affiliations:** 1 Water Conservancy College, North China University of Water Resources and Electric Power, Zhengzhou, 450046 China; 2 Collaborative Innovation Center of Water Resources Efficient Utilization and Protection Engineering, Zhengzhou, 450046 China; 3 Technology Research Center of Water Conservancy and Marine Traffic Engineering, Zhengzhou, 450046 Henan Province China

**Keywords:** Environmental sciences, Hydrology

## Abstract

Accurate medium and long-term runoff forecasts play a vital role in guiding the rational exploitation of water resources and improving the overall efficiency of water resources use. Machine learning is becoming a common trend in time series forecasting research. Least squares support vector machine (LSSVM) and grey model (GM(1,1)) have received much attention in predicting rainfall and runoff in the last two years. “Decomposition-forecasting” has become one of the most important methods for forecasting time series data. Complete ensemble empirical mode decomposition with adaptive noise (CEEMDAN) decomposition method has powerful advantages in dealing with nonlinear data. Least squares support vector machine (LSSVM) has strong nonlinear fitting ability and good robustness. Gray model (GM(1,1)) can solve the problems of little historical data and low serial integrity and reliability. Based on their respective advantages, a combined CEEMDAN–LSSVM–GM(1,1) model was developed and applied to the runoff prediction of the lower Yellow River. To verify the reliability of the model, the prediction results were compared with the single LSSVM model, the CEEMDAN–LSSVM model and the CEEMDAN–support vector machines (SVM)–GM(1,1). The results show that the combined CEEMDAN–LSSVM–GM(1,1) model has a high accuracy and the prediction results are better than other models, which provides an effective prediction method for regional medium and long-term runoff prediction and has good application prospects.

## Introduction

Runoff prediction is an important element in hydrological forecasting research, and its prediction results can provide the basis for flood and drought prevention, reservoir scheduling and hydroelectric power generation^1,2^. Because the time interval between medium and long-term forecasts is too long and there are too many uncertainties, the medium and long-term runoff series are highly nonlinear and random, and the forecast results are not ideal^3^. How to establish a runoff prediction model with higher forecast accuracy is extremely important for the optimal allocation of water resources in the basin and regional development planning.

Currently, medium and long-term runoff prediction mainly includes process-driven model and data-driven model^4^. Process-driven models rely on the scientific theories of hydrology, hydraulics and erosion dynamics^5^, consider the physical mechanisms within the water cycle system in all aspects, and integrate factors such as land use, soil type, meteorological changes, and water quality advantages and disadvantages to simulate the runoff production process, with the SWAT model^6^, the DHSVM model^7^, and Xin'anjiang model^8^ as representatives. Since the rainfall runoff process is influenced by various factors such as topography, rainfall distribution, soil properties, land use, and climate change, process-driven models require a large amount of data for modeling, and insufficient data will have an impact on the successful establishment of the model, while data-driven models require little data information and have a fast development time, so the data-driven approach is still mainly used for medium and long-term runoff prediction^9,10^. Data-driven models target the optimal relationship between data and use mathematical methods to establish a relationship between the input data and the output target of the model. Traditional data-driven models include multiple regression models, time series models, mathematical statistical models, etc. With the development of computer theory, modern data-driven models make more use of neural networks, fuzzy mathematics, gray systems and support vector machines for hydrological data prediction^11^. Typically, data-driven models can be further classified into three categories: statistical-based predictive models, machine learning models, and combinatorial models. Thomas et al.^12^ proposed an autoregressive model (AR). Carlson et al.^13^ applied an autoregressive moving average (ARMA) model to annual runoff predictions. Elshorbagy et al.^14^ applied a multiple linear regression model (MLR) for daily runoff prediction. The above statistical modeling methods are based on linear regression theory, and the prediction accuracy still needs to be improved when dealing with complex runoff information, and more powerful models are needed for runoff prediction to deal with non-linear and complex runoff simulations^15^. Cortes et al.^16^ proposed a support vector machine model (SVM) based on the Vapnik–Chervonenkis dimensionality theory and structural risk minimization theory in statistical theory. Liao et al.^17^ attempted to apply SVM models to the field of runoff prediction and compared them with the threshold regression TR model to confirm the superiority of SVM models in runoff prediction. Li et al.^18^ proposed a support vector machine model based on the principle of least squares (LSSVM), and experimentally confirmed that the runoff prediction results of the LSSVM model can still maintain high accuracy when the sample data are small. Shabri et al.^19^ applied the cross-validation method and grid search method to the LSSVM model for the selection of parameters in the runoff prediction process, which greatly reduced the modeling time of the LSSVM model.

However, due to the high nonlinearity of runoff series, relying on raw data to build machine learning models may not meet the prediction needs. Based on the research on machine learning models, scholars at home and abroad have conducted extensive research on improving the accuracy of runoff prediction by pre-processing time series. The prediction process can be simplified by pre-noise reduction or decomposition of the data, and the non-linear and non-smooth characteristics in the hydrological series can be analyzed in advance before the prediction, which can effectively improve the efficiency of the later prediction. Mallat^20^ proposed an easy to implement and less computational multi scale analysis algorithm, which processes the raw data into multiple layers of smoother low-frequency components and high-frequency components, which can improve the prediction accuracy of the raw time series data. Huang et al.^21^ proposed the empirical mode decomposition (EMD), which is a method for the analytical processing of nonlinear and non-smooth signals. The original complex nonlinear signal is decomposed by EMD into several intrinsic mode functions (IMF) and a residual. And the IMF obtained by the EMD method may have the problem of mode mixing. Wu et al.^22^ proposed the ensemble empirical mode decomposition (EEMD) method, which adds Gaussian white noise to the original signal during the decomposition process and can effectively suppress mode mixing. Complete ensemble empirical mode decomposition with adaptive noise (CEEMDAN) decomposition of adaptive noise adds a finite number of adaptive white noises at each stage, reducing the number of iterations and improving reconstruction accuracy compared to EEMD^23^. Dragomiretskiy et al.^24^ proposed the Variational mode decomposition (VMD) method. The method is an adaptive, fully non-recursive approach to modal variation and signal processing. Effective separation of intrinsic modal functions (IMF) and frequency domain division of signals can be achieved. The effective decomposition components of the given signal are obtained, and finally the optimal solution of the variational problem is obtained. Raj Huan et al.^25^ proposed an EEMD–LSSVM model for predicting dissolved oxygen data based on time series and compared the prediction results with a single LSSVM model, and the results showed that the EEMD–LSSVM model has high prediction accuracy and generalization ability. Huang et al.^26^ applied the EMD–GM(1,1) model to predict landslide deformation and compared it with the traditional GM(1,1) model, and found that EMD–GM(1,1) had higher prediction accuracy. Jamei et al.^27^ used the Multivariate Variational Mode Decomposition (MVMD) decomposition method with the LSSVM model applied to predict the daily wave energy in coastal areas, this research can help authorities in the field of renewable and sustainable energy for better planning and development. Jamei et al.^28^ used a novel decomposition method, namely time varying filter-based empirical mode decomposition (TVF–EMD), before predicting daily flood levels at two sites in the Clarence River, Australia. Raj et al.^29^ studied the sea level prediction problem for small island countries such as Kiribati and Tuvalu, and he used a new method of data decomposition, namely, continuous variational modal decomposition (SVMD).

The water resources of the Yellow River basin are facing the problems of large dynamic changes in river runoff hydrology, uneven seasonal distribution, and large sand content of rivers. The source of the Yellow River has a decreasing trend of historical flood runoff under the influence of climate change and human activities, and the middle and lower reaches of the Yellow River are in the temperate monsoon zone with large seasonal variation of precipitation, especially under the influence of global warming, which has increased the dynamic variability of natural runoff of the Yellow River. Therefore, it is particularly necessary to study the prediction of runoff in the Yellow River basin, especially in the densely populated areas of the middle and lower reaches of the Yellow River. Timely and accurate prediction of natural runoff in the middle and lower reaches of the Yellow River will provide a basis for promoting the rational and optimal allocation of water resources in the Yellow River area, maximizing the demand for water for agriculture, industry and domestic use, and promoting the virtuous cycle of the regional ecosystem, as well as providing a basis for rational allocation and development of water scheduling implementation plans. Based on the good decomposition effect of CEEMDAN and the combination effect with LSSVM model and GM(1,1), a CEEMDAN–LSSVM–GM(1,1) model is constructed in this paper to predict monthly runoff from four hydrological stations in the lower Yellow River, and the prediction results are compared with those of several different models to illustrate the effectiveness of the model and provide a new combined machine learning model solution to the runoff prediction problem.

## Methods

### CEEMDAN

**Figure 1:**
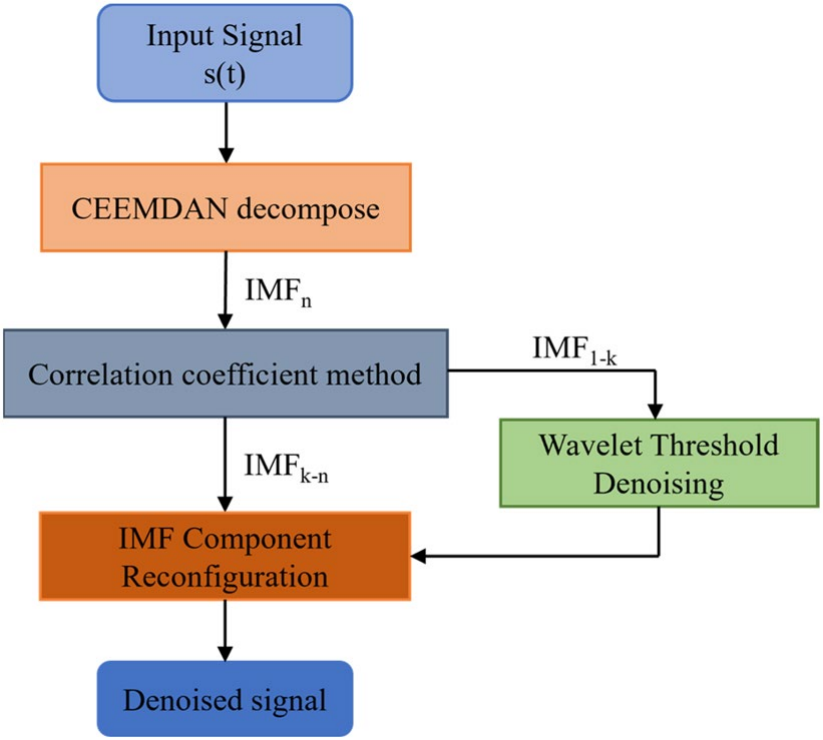
CEEMDAN operating principle.

The Complete Ensemble Empirical Mode Decomposition with Adaptive Noise (CEEMDAN) method is an improved EMD algorithm, which overcomes the modal confusion problem of the original EMD method and can more accurately extract from the nonlinear sequences the relatively smooth Intrinsic Mode Functions (IMF) and Residuals (Res) of these components of the nonlinearity decreases layer by layer^30^, which can clearly reflect the fluctuation characteristics of different cycles and provide convenience for the analysis and prediction of complex sequences. Figure 1 shows the working principle of CEEMDAN.

The runoff time series is denoted by $S(t)$ and $V^{i} (t)$ is a Gaussian white noise series with standard normal distribution added in the $i$th trial, so the $i{\text{th}}$ signal series can be expressed as Eq. (1):

1 $$ S^{i} (t) = S(t) + \varepsilon_{0} V^{i} (t)\quad i \in \{ 1,2, \ldots ,M\} $$

where $\varepsilon_{0}$ is the noise factor, $M$ is the number of integrations, generally between 10 and 20.

Define the operator $emd_{i} ( \, )$ as the modal component of the $i{\text{th}}$ stage generated by applying the EMD algorithm, and the $i{\text{th}}$ modal component obtained after decomposition with the CEEMDAN algorithm is noted as $IMF_{i}$.

The specific steps of the algorithm are as follows:

1. A series of adaptive Gaussian white noise $\varepsilon_{0} V^{i} (t)\quad i \in \{ 1,2, \ldots ,M\}$ is first added to the original time series $S(t)$, $M$ trials are performed for the $i{\text{th}}$ signal $S^{i} (t)$, and the new time series $S^{i} (t)$ is decomposed using the EMD algorithm, and the first IMF component $IMF_{1}^{i} (t)$ obtained from the decomposition is averaged to obtain the first CEEMDAN modal component.
  2 $$ IMF_{1} (t) = \frac{1}{M}\mathop \sum \limits_{i = 1}^{M} IMF_{1}^{i} (t) $$
2. The first component $IMF_{1} (t)$ of the decomposition is removed from the original time series $S(t)$ to obtain the first residual series $R_{1} (t)$.
  3 $$ R_{1} (t) = S(t) - IMF_{1} (n) $$
3. In the same way as in step (1), a series of adaptive Gaussian white noise $\varepsilon_{1} V^{i} (t)\quad i \in \{ 1,2, \ldots ,M\}$ is added to the first residual sequence, and the EMD decomposition of the sequence $R_{1} (t) + \varepsilon_{1} emd_{1} \left( {V^{i} (t)} \right)$ is continued to obtain the second IMF modal component.
  4 $$ IMF_{2} (t) = \frac{1}{M}\mathop \sum \limits_{i = 1}^{M} emd_{1} \left( {R_{1} (t) + \varepsilon_{1} emd_{1} \left( {V^{i} (t)} \right)} \right) $$
4. Repeat steps (1) and (2) above to calculate the $k{\text{th}}$ residual signal and $k + 1$ modal components for each of the remaining stages to obtain the remaining IMF modal components, where $K$ is the total number of IMF modes.
  5 $$ R_{k} (t) = R_{k - 1} (t) - IMF_{k} (t)\quad (k = 2,3, \ldots K) $$
  6 $$ IMF_{(k + 1)} (t) = \frac{1}{M}\mathop \sum \limits_{i = 1}^{M} emd_{1} \left( {R_{k} (t) + \varepsilon_{k} emd_{k} \left( {\nu^{i} (t)} \right)} \right) $$
5. The termination condition of the decomposition is that the residual sequence cannot be decomposed further if there are at most 2 residual extreme value points. The remaining residual sequence that cannot be decomposed further is called the residual signal, and its expression is:
  7 $$ r(t) = S(t) - \mathop \sum \limits_{k = 1}^{K} IMF_{k} (t) $$
6. Therefore, the original time series $S(t)$ can be expressed by Eq. (8) after CEEMDAN decomposition:
  8 $$ S(n) = r(n) + \mathop \sum \limits_{k = 1}^{K} IMF_{k} (n) $$

Based on the above process we can see that the CEEMDAN decomposition is able to perform an accurate reconstruction of the original signal data. The problem of mode mixing in EMD decomposition is avoided in the whole implementation of the algorithm, and at the same time effectively reduces the number of iterations compared to the EEMD decomposition to increase the reconstruction accuracy and improve its computational efficiency, which is more suitable for the analysis of non-linear signals.

### LSSVM

Least squares support vector machine (LSSVM) is a special kind of support vector machine, the basic idea is to map nonlinear data to linear regression in high-dimensional space^31^, the main algorithm is to use least squares to transform the inequality constraints of the actual problem into a problem of solving a set of linear equations, which simplifies the calculation. With the research and development, it has been widely used in the field of hydrology, such as hydrological series prediction model, basin annual and monthly runoff prediction, etc., and has achieved better results.

The derivation process for the LSSVM algorithm is as follows:

1. Given a training sample set of ${\text{P}} = \left\{ {\left( {x_{k} ,y_{k} } \right),k = 1,2, \ldots N} \right\}$, where $x_{{\text{k}}} \in R^{n}$, ${\text{y}}_{{\text{k}}} \in R$. The LSSVM algorithm non-linear regression function is:
  9 $$ f(x) = \omega^{T} \varphi (x) + b $$
  where $b$ is the deviation value; $\omega$ is the weight vector.
2. By means of a non-linear transformation, the optimal hyperplane solution in higher dimensions can be transformed into the following form.
  10 $$ \left\{ {\begin{array}{*{20}l} {\min J(\omega ,b,\xi ) = \frac{1}{2}\omega^{T} \omega + \frac{c}{2}\mathop \sum \limits_{i = 1}^{N} \xi_{k}^{2} } \hfill \\ { \, s.t.y_{k} = \omega^{T} \varphi \left( {x_{k} } \right) + b + e_{k} } \hfill \\ \end{array} } \right. $$
  where $J$ is the loss function, $c$ is the penalty factor, $\xi_{k}$ is the error, and $\varphi \left( {x_{k} } \right)$ is a non-linear function.
3. Constructing Lagrangian functions.:
  11 $$ L(\omega ,b,e,\alpha ) = J(\omega ,b,e) - \mathop \sum \limits_{k = 1}^{N} \alpha_{k} \left\{ {\omega^{T} \phi \left( {x_{k} } \right) + b + \xi_{k} - y_{k} } \right\} $$
4. Then, according to the optimization condition, find the partial derivatives of $\omega ,b,e,\alpha$ respectively, and make the partial derivatives equal to 0. We have:
  12 $$ \left\{ {\begin{array}{*{20}l} {\frac{\partial L}{{\partial \omega }} = 0 \Rightarrow \omega = \sum\limits_{i = 1}^{n} {\alpha_{i} } y_{i} \varphi \left( {x_{i} } \right)} \hfill \\ {\frac{\partial L}{{\partial b}} = 0 \Rightarrow \sum\limits_{i = 1}^{n} {\alpha_{i} } = 0} \hfill \\ {\frac{\partial L}{{\partial e_{i} }} = 0 \Rightarrow \alpha_{i} = \gamma e_{i} } \hfill \\ {\frac{\partial L}{{\partial \alpha_{i} }} = 0 \Rightarrow \omega^{T} \varphi \left( {x_{i} } \right) + b + e_{i} - y_{i} = 0} \hfill \\ \end{array} } \right. $$
5. By eliminating $\omega$ and $\xi_{i}$, the following linear system is obtained by simplification:
  13 $$ \left[ {\begin{array}{*{20}c} 0 & {I^{T} } \\ I & {{\Omega } + I/C} \\ \end{array} } \right]\left[ {\begin{array}{*{20}l} b \hfill \\ a \hfill \\ \end{array} } \right] = \left[ {\begin{array}{*{20}l} 0 \hfill \\ y \hfill \\ \end{array} } \right] $$
  where $y = \left[ {y_{1} , \ldots ,y_{n} } \right]^{{\text{T}}}$, ${\text{I}} = [1, \ldots ,1]^{{\text{T}}}$, $a = \left[ {a_{1} , \ldots ,a_{n} } \right]^{{\text{T}}}$; ${\Omega }_{M \times M}$ is the kernel matrix and the radial basis function RBF is the kernel function, then:
  14 $$ {\Omega }_{ij} = K\left( {x_{i} ,x_{j} } \right) = \varphi \left( {x_{i} } \right)^{T} \varphi \left( {x_{j} } \right) $$
6. After finding $a$ and $b$ from Eq. (13), the expression for the LSSVM non-linear regression function is obtained as
  15 $$ y = \omega^{T} \phi \left( {x_{k} } \right) + b = \mathop \sum \limits_{k = 1}^{N} a_{k} K\left( {x,x_{k} } \right) + b $$

Compared with SVM, LSSVM has smaller computational complexity and therefore faster processing speed; when dealing with dynamic problems, LSSVM can be extended into an autoregressive form; strong non-linear fitting ability, sparsity, generalization ability, good robustness and the ability to find optimal solutions quickly.

### GM(1,1)

The grey model (GM) is a system that includes partly unknown and partly known information^32^, and grey theory is a mathematical method for solving systems with incomplete information by replacing the original random process with a grey process that has a temporal pattern and a limited range of variation, thus transforming the initial data, where no pattern can be found, into data that is easy to study with regular variation. The theory can therefore solve the problem of uncertainty in situations where there is insufficient information and too little data. The modelling steps of the model are as follows: Let $X^{(0)}$ be the original time series:

16 $$ X^{(0)} = \left[ {X^{(0)} (1),X^{(0)} (2), \ldots ,X^{(0)} (n)} \right],\quad i = 1,2, \ldots ,n $$

1. The original time series is generated cumulatively once to obtain the generated series $X^{(1)}$. This weakens the randomness and enhances the regularity, and the generated series will be close to the regularity of the exponential relationship.
  17 $$ X^{(1)} = \left[ {X^{(1)} (1),X^{(1)} (2), \ldots ,X^{(1)} (n)} \right] $$
  where
  18 $$ X^{(1)} (k) = \mathop \sum \limits_{i = 1}^{k} X^{(0)} (i),k = 1,2, \ldots n $$
2. The cumulative sequence $X^{(1)}$ is generated by making the nearest neighbor mean according to Eq. (18) to obtain the sequence $Z^{(1)}$
  19 $$ Z^{(1)} (k) = \frac{1}{2}X^{(1)} (k) + \frac{1}{2}X^{(1)} (k - 1),k = 1,2, \ldots n $$
  20 $$ Z^{(1)} = \left[ {Z^{(1)} (1),Z^{(1)} (2), \ldots Z^{(1)} (n)} \right] $$
3. The differential equation for GM(1,1) is established from the cumulative generating sequence $X^{(1)}$.
  21 $$ \left\{ {\begin{array}{*{20}l} {\frac{{dX^{(1)} }}{dk} + aX^{(1)} = b} \hfill \\ {X^{(1)} (1) = X^{(0)} (1)} \hfill \\ \end{array} } \right. $$
  where $a$ is the development coefficient and $b$ is the amount of grey action. From the solution method in the theory of ordinary differential equations, the analytical solution of the equation is found as:
  22 $$ \hat{X}^{(1)} (k + 1) = \left[ {X^{(0)} (1) - \frac{b}{a}} \right]e^{ - ak} + \frac{b}{a},\quad (k = 1,2, \ldots ,n) $$
4. Find the values of $a$, $b$ by the principle of least squares.
  23 $$ \overline{a} = (a,b)^{T} = \left( {B^{T} B} \right)^{ - 1} B^{T} Y $$
  where
  24 $$ Y = \left[ {\begin{array}{*{20}c} {X^{(0)} (2)} \\ {X^{(0)} (3)} \\ \vdots \\ {X^{(0)} (n)} \\ \end{array} } \right] $$
  25 $$ B = \left[ {\begin{array}{*{20}c} { - Z^{(1)} (2)} & 1 \\ { - Z^{(1)} (3)} & 1 \\ \vdots & \vdots \\ { - Z^{(1)} (n)} & 1 \\ \end{array} } \right] $$
5. Reducing Eq. (22) to a grey forecasting model for the original time series $X^{(0)}$.
  26 $$ \hat{X}^{(0)} (k + 1) = \left( {1 - e^{a} } \right)\left[ {X^{(0)} (1) - \frac{b}{a}} \right]e^{ - ak} ,(k = 1,2, \ldots ,n) $$

The grey model is a differential equation model created by generating new data from the original data series. Since the solution of its differential equation is in exponential form, it is more accurate in predicting variables with exponential growth or decreasing trends. Although the model is suitable for modelling sequences that are monotonic and vary exponentially, the non-linear and non-smooth nature of the monthly runoff series makes it so that the generated series after the accumulation of the original runoff series does not necessarily obey the exponential variation law, so this section is based on the residuals obtained from the CEEMDAN decomposition for forecasting, which is feasible with this model as the residuals has a monotonic downward trend.

### Model development

The partitioning of time series datasets is often judged according to the rule of thumb; normally, the training part of the dataset should carry more than 60% of the overall and the validation part should be more than 20% of the overall, and many researchers have used different partitioning situations: Kumar et al.^33^ used 70% of the data to train RNN and LSTM models for the “all-India” monthly average precipitation data to build the model; Liu et al.^34^ used 80% of the data as the training set to train the model for wind speed prediction and the remaining 20% portion as the test set. We prevent overfitting by increasing the training data of the model. In this study, 90% of the data were taken to train the model and 10% of the data were used to test the model performance.

### CEEMDAN–LSSVM–GM(1,1) model

**Figure 2:**
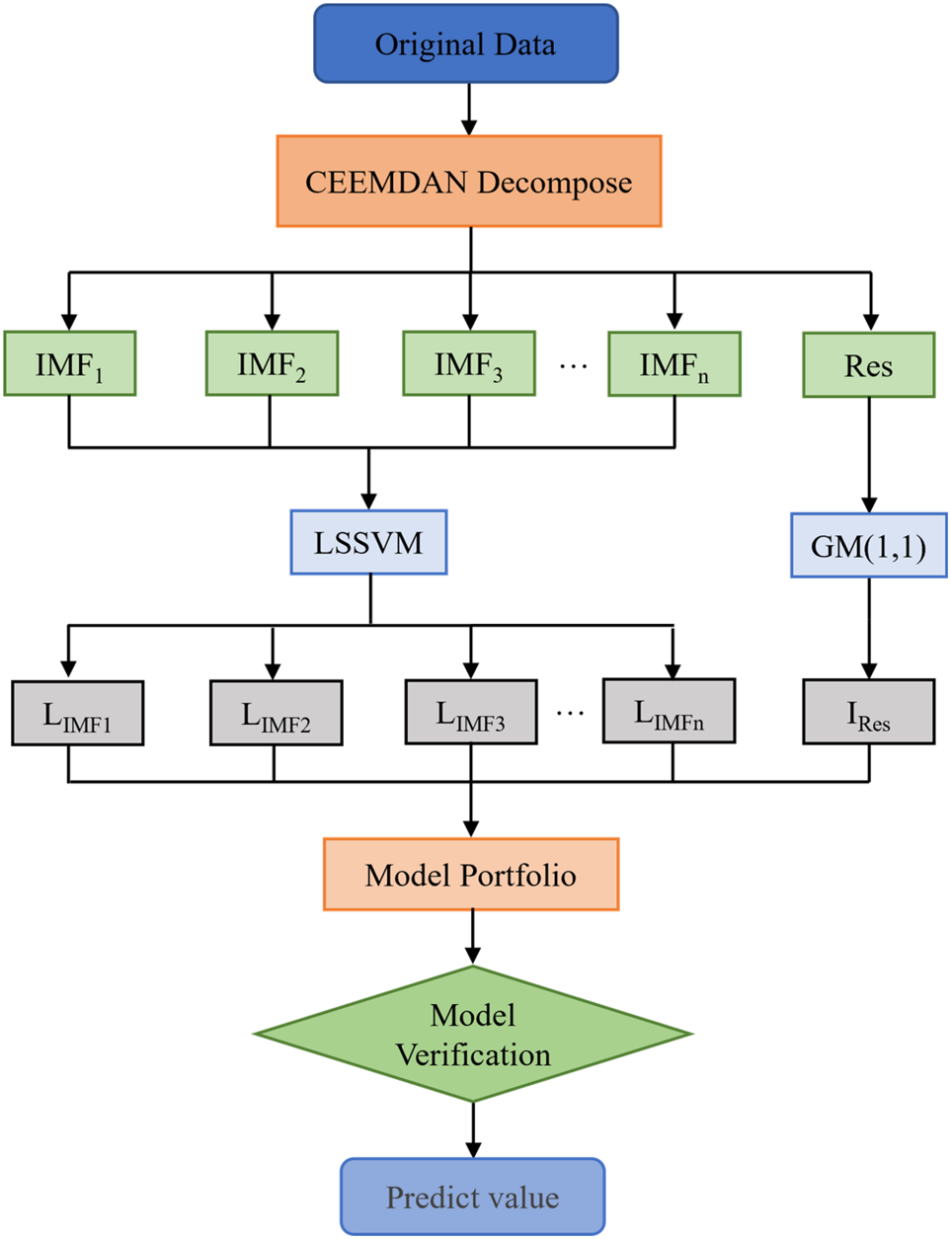
Flow chart of CEEMDAN–LSSVM–GM(1,1) model.

In this paper, the monthly runoff data of four hydrological stations in the lower reaches of the Yellow River: Huayuankou Station, Gaocun Station, Aishan Station and Lijin Station from 1965 to 2014 were selected for the study, and the runoff characteristics and change patterns were analyzed and examined, and on this basis, a runoff prediction model was established using least squares support vector machine and grey theory, and the flow chart is shown in Fig. 2.

1. Monthly historical runoff data of four hydrological stations in the lower Yellow River from 1965 to 2014 were decomposed using CEEMDAN to obtain several high-frequency IMF components and a low-frequency residual.
2. A total of 550 months of data from January 1965 to October 2010 were used for training the model, and 50 months of data from November 2010 to December 2014 were used for validation.
3. The high-frequency IMF components obtained from the CEEMDAN decomposition are predicted using the LSSVM model, and then the low-frequency residuals are predicted using the GM(1,1) model.
4. Weighted summation of the predictions of the IMF components and residuals calculated in step (3) to obtain the final monthly runoff time series prediction results.

### Model evaluation indicators

In order to verify the prediction results of the CEEMDAN–LSSVM–GM(1,1) model for the monthly runoff at four hydrological stations in the lower reaches of the Yellow River, the following four evaluation indicators were used to evaluate the prediction results, with Nash–Sutcliffe efficiency coefficient (NSE), mean absolute error (MAE), mean absolute percentage error (MAPE), and root mean squared error (RMSE) as quantitative evaluation criteria, and the calculation equations are as follows:

27 $$ NSE = 1 - \frac{{\sum\limits_{i = 1}^{n} {(y_{i} - \hat{y}_{i} )^{2} } }}{{\sum\limits_{i = 1}^{n} {\left( {y_{i} - \overline{y}} \right)^{2} } }},i = 1,2, \ldots ,n $$

28 $$ MAE = \frac{1}{N}\mathop \sum \limits_{i = 1}^{{\text{n}}} \left| {\left( {y_{i} - \hat{y}_{i} } \right)} \right|,\;\;i = 1,2, \ldots ,n $$

29 $$ \, MAPE \, = \frac{1}{N}\sum\limits_{i = 1}^{N} {\left| {\frac{{y_{i} - \hat{y}_{i} }}{{y_{i} }}} \right|} \times 100\% ,\;\;i = 1,2, \ldots ,n $$

30 $$ RMSE = \sqrt {\frac{1}{N}\sum\limits_{i = 1}^{N} {\left( {y_{i} - \hat{y}_{i} } \right)^{2} ,\;\;i = 1,2, \ldots ,n} } $$

where $\hat{y}_{i}$ and $y_{i}$ represent the predicted runoff series and its corresponding original runoff series respectively, $\overline{y}$ is the monthly average runoff volume of the original runoff series, and $n$ is the number of runoff series values.

## Case study

### Study area

**Figure 3:**
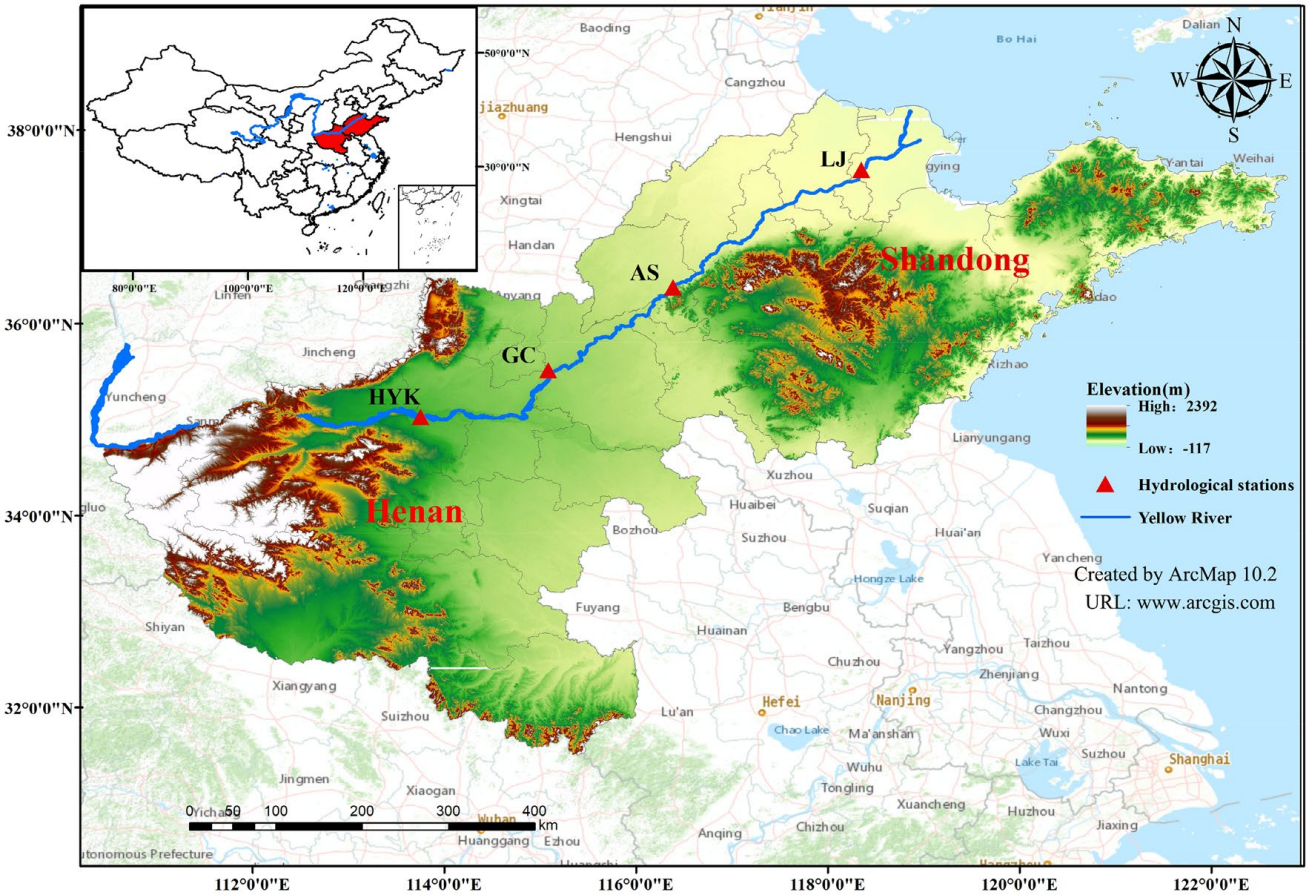
Hydrographic station distribution map.

**Figure 4:**
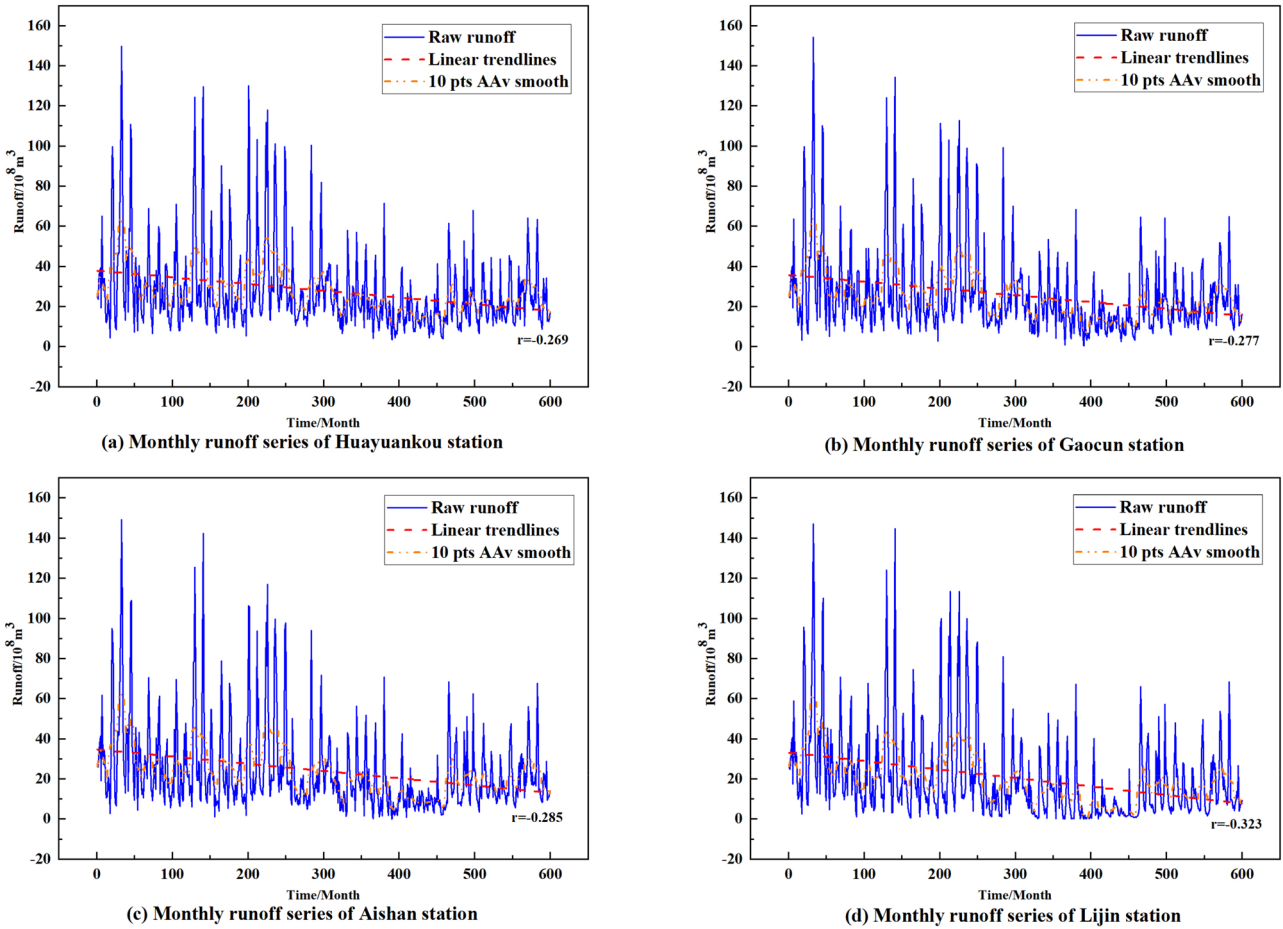
The course of monthly runoff at each hydrological station 1965–2014.

The Yellow River basin is located in the mid-latitude zone, with a range of 95° 53′–119° 05′ E, 32° 10′– 42° 50′ N. The Yellow River originates from the Bayankara Mountains on the Qinghai-Tibet Plateau in Qinghai Province, and spans a large area from east to west, flowing from west to east through Qinghai, Sichuan, Gansu, Ningxia, Inner Mongolia, Shaanxi, Shanxi, Henan and Shandong. The Yellow River has a total length of 5464 km, with an east–west direction of about 1900 km and a north–south direction of about 1100 km, covering a total area of 752,000 km^2^ and a large geographical span. Three-quarters of the Yellow River basin is in the arid and semi-arid zone, with a predominantly continental monsoon climate. The average annual evaporation in the Yellow River basin ranges from 700 to 1800 mm, with high evaporation. There are many tributaries in the Yellow River basin and the spatial and temporal distribution of runoff is uneven and seasonally variable^35^. The Huayuankou hydrological station is located in Zhengzhou City, Henan Province, while the Gaocun hydrological station, Aishan hydrological station and Lijin hydrological station are located in Heze City, Liaocheng City and Dongying City, Shandong Province respectively. These four hydrological stations are responsible for the important tasks of water resources utilisation in the lower reaches of the Yellow River, regional water resources development and the investigation of hydrological and water resources change patterns, and have well preserved hydrological data. Runoff data from all hydrological stations in the study area are available in the Water Information System of the Yellow River Network (www.yrcc.gov.cn). The geographical location map of the study area is shown in Fig. 3, it is created using ArcMap 10.2, URL:www.arcgis.com, and the runoff series from 1965 to 2014 for the four hydrological stations are shown in Fig. 4.

### Data sources

Linear regression analysis and moving average were selected to analyze the trend of monthly runoff at the four hydrological stations from 1965 to 2014. Figure 4 shows the process of runoff at each station. It can be seen that the monthly runoff at the four hydrological stations in the lower reaches of the Yellow River is highly non-linear and non-stationary, with the extreme values of monthly runoff occurring mostly during the flood season, and showing a high degree of time-variability and complexity. The magnitude and variability of monthly runoff at the Huayuankou hydrological station is higher than at the other three stations due to its special geographical location. The linear trend line and the periodic moving average curve in the graph represent the trend of monthly runoff. This indicates that the runoff shortage problem in the lower Yellow River has become increasingly serious, and that more accurate prediction models are needed to provide reliable and stable predictions of runoff.

**Figure 5:**
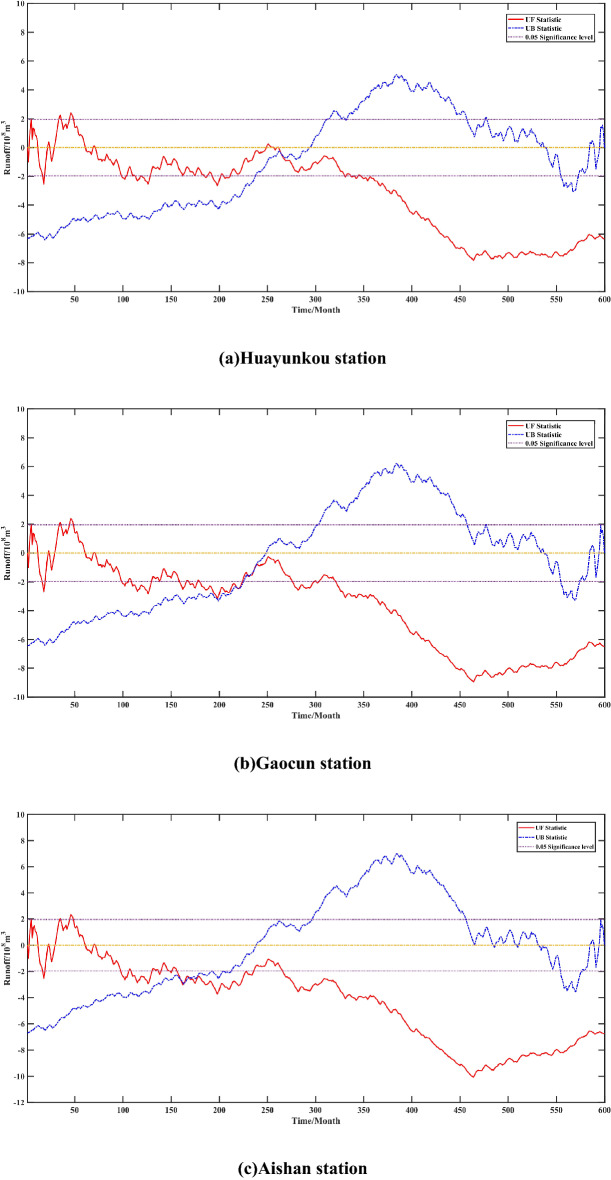

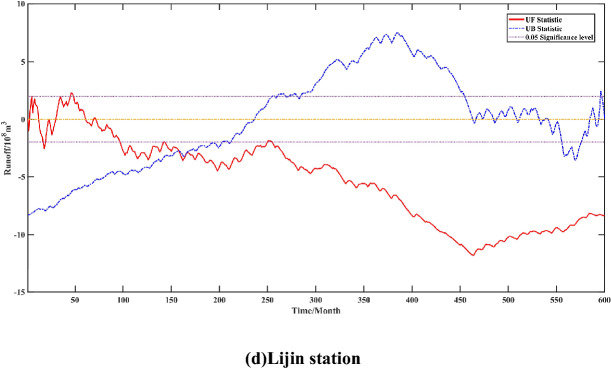
Mann–Kendall test for runoff from January 1965 to December 2014 at each station.

Mutation detection of hydrological data helps in hydrological data analysis and hydrological data prediction. Reliable mutation detection can analyze the stage change characteristics of hydrological data and find out the factors that affect the hydrological prediction effect. It is of great guiding significance for in-depth analysis of hydrological data change characteristics and improvement of hydrological data prediction effect^36^. Given a significant level $\alpha = 0.05$, the critical value $u_{0.05} = \pm 1.96$, and the results of the Mann–Kendall test were obtained as shown in Fig. 5. The UF values corresponding to the intersection of the UF and UB statistics at the four hydrological stations are all less than 0, indicating that these intersection points are the abrupt change points for the reduction of monthly mean runoff, with the intersection of the UF and UB statistics at the Huayuankou hydrological station lying within the critical interval, indicating a significant abrupt change at this point, and the intersection of the UF and UB statistics at the remaining three stations all lying outside the critical interval, indicating that no significant abrupt change in runoff has occurred at these stations.

**Figure 6:**
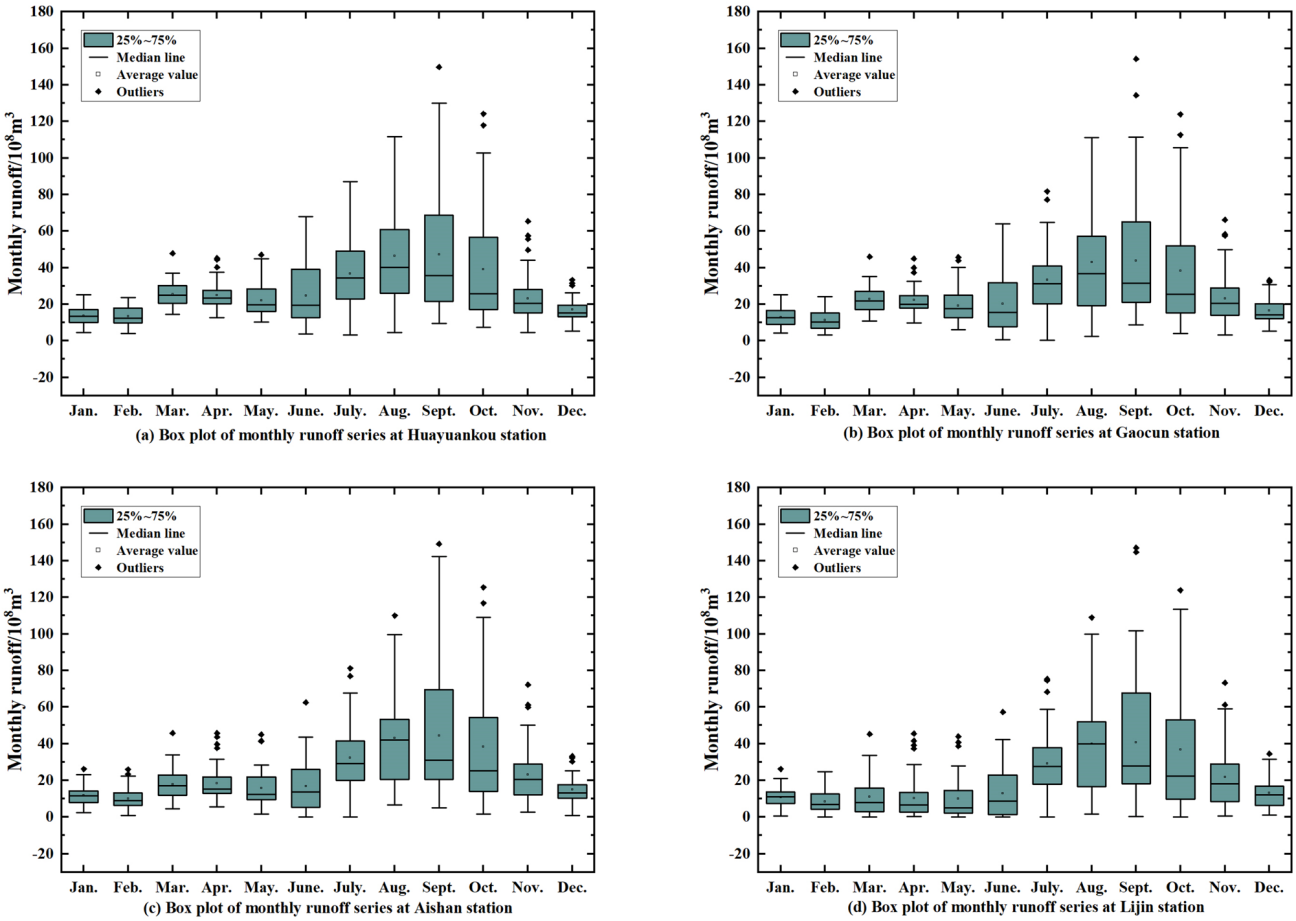
Yellow River downstream runoff box diagram.

In this paper, a total of 600 months of hydrological data from January 1965 to December 2014 were collected from four hydrological stations in the lower Yellow River, of which 550 months of runoff data from January 1965 to December 2009 were used as the training set data, and 50 months of runoff data from January 2010 to December 2014 were used as the test set to validate the model effect. Box plots were used to detect anomalies in the runoff data. Figure 6 shows the results of the box plot identification of the monthly runoff data of the lower Yellow River over the years. It can be seen from the figure that the box corresponding to June–October in the lower Yellow River is longer, which indicates that the runoff volume fluctuates more drastically in these months, among which, there are more anomalous values in July. The reason for the above phenomenon may be that this period is the flood season of the Yellow River basin, which is greatly influenced by rainfall as well as certain extreme climatic factors, and the runoff volume accumulates in a short period of time, showing an irregular change in magnitude. Therefore, normalisation of the decomposed monthly runoff data is considered to reduce the volatility of the raw runoff data and enhance the stability of the model prediction. The normalisation formula is as follows:

31 $$ y_{i}^{\prime } = \frac{{y_{i} - \min \left( {y_{i} } \right)}}{{\max \left( {y_{i} } \right) - \min \left( {y_{i} } \right)}} $$

## Results and discussion

### Results

**Figure 7:**
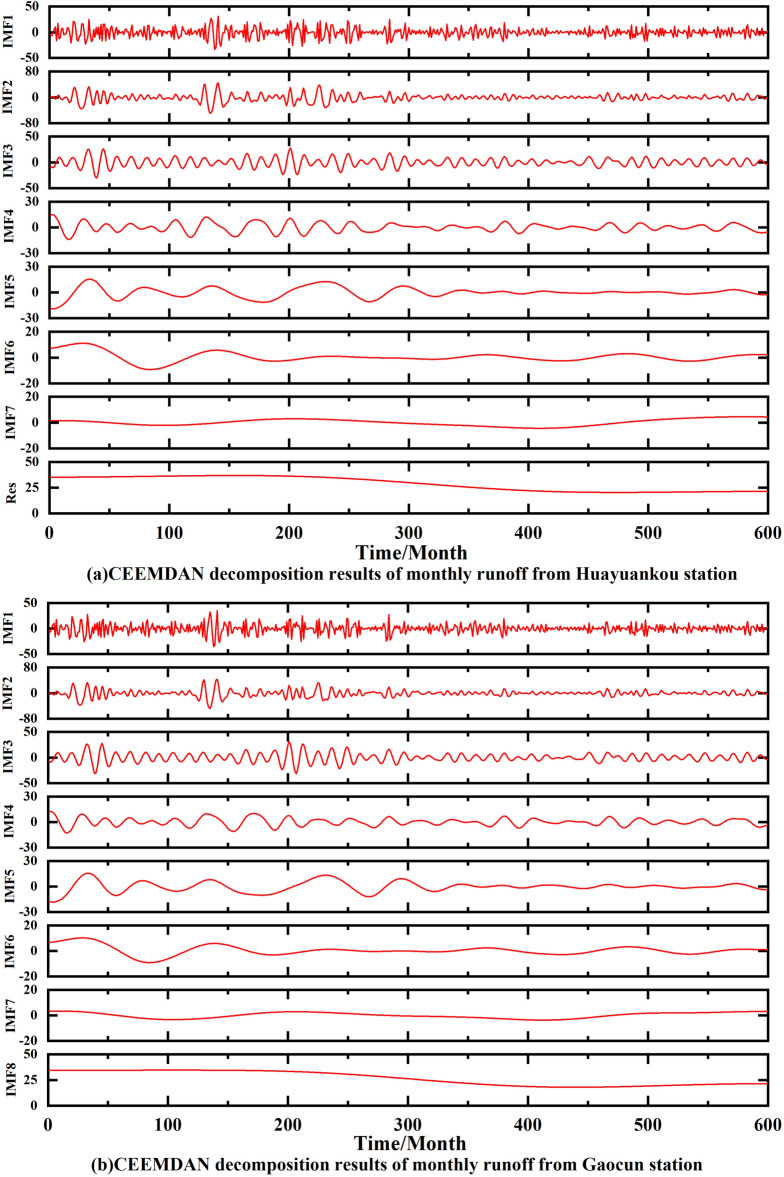

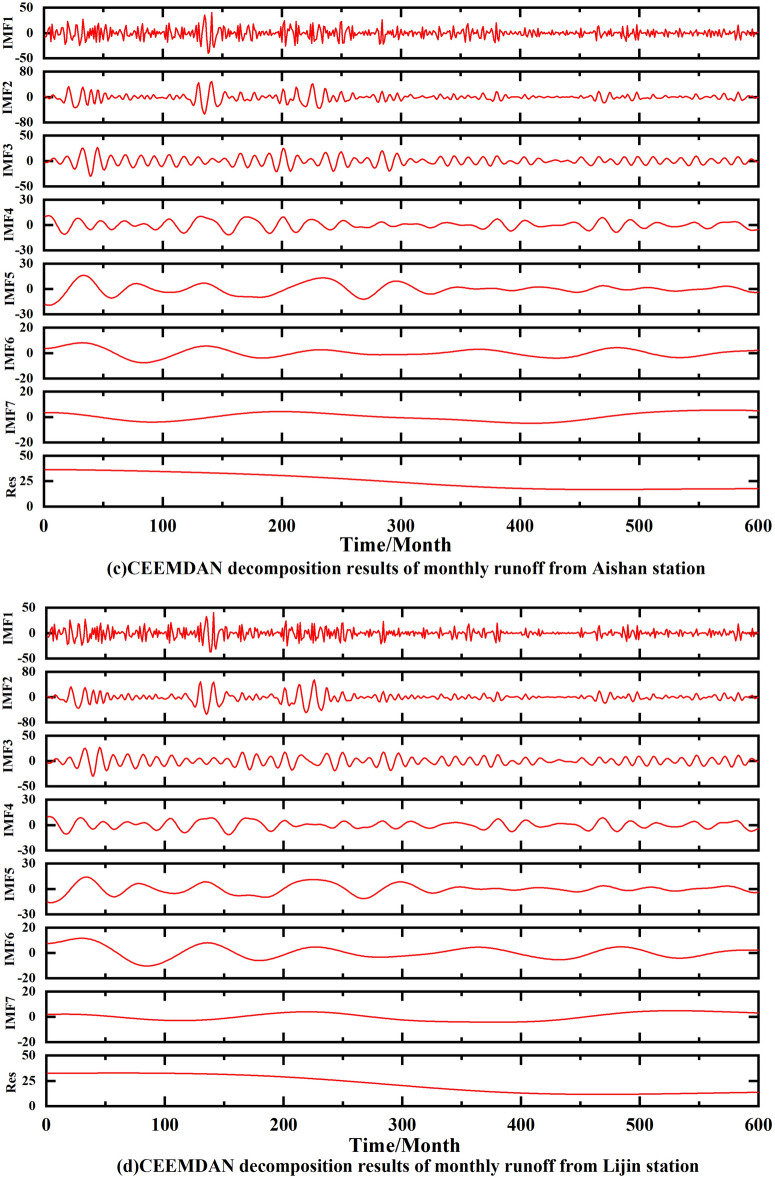
Monthly runoff data from the lower Yellow River stations using CEEMDAN decomposition.

The results obtained after decomposing the monthly runoff data from the four hydrological stations using CEEMDAN are shown in Fig. 7. It can be seen that the IMF1 frequency is the highest, the amplitude is the largest, and the wavelength is the shortest, while the periodicity is the smallest at the same time, at the Huayuankou station. IMF1 to the rest of the frequency gradually decreases, the amplitude decreases and the periodicity becomes stronger. The stability of IMF2–IMF7 gradually increases, representing different time-scale components of the original runoff, while retaining some periodic features and some trends of the original runoff. The amplitude of IMF1 at Gaocun station is the largest, and IMF2–IMF7 gradually stabilize. The amplitude of IMF1 at Aishan station is also the largest, and IMF2–IMF7 fluctuates more from 1965 to 1982 and gradually decreases from 1983 to 2014. The frequency of IMF1 is the highest in Lijin station. While the fluctuations of IMF2–IMF7 from 1965 to 1990 are more obvious, the fluctuations from 1991 to 2014 are more stable. Res is the residual of the original runoff, which represents the overall trend of the original runoff series and is an important criterion to judge the change pattern of runoff. From the change curve of Res in Fig. 7, it can be seen that the runoff from Huayuankou and Gaocun stations gradually increased from 1965 to 1982 and decreased from 1983 to 2014; the runoff from Aishan and Lijin stations gradually increased from 1965 to 1974 and decreased from 1975 to 2014. The above observations show that the general trend of runoff in the lower Yellow River area has been increasing and then decreasing in the last 60 years.

After preprocessing the raw runoff time series, the LSSVM and GM(1,1) models were used to predict the high-frequency IMF components and low-frequency trend terms, respectively. The parameters of the LSSVM model are set as follows: the radial basis function (RBF) is chosen as the kernel function, and its expression is:$K\left( {x_{i} ,x_{j} } \right) = \exp \left( { - g\parallel x_{i} - x_{j} \parallel^{2} } \right)$, The size of the penalty factor *c* and the kernel function parameter *g* affect the prediction accuracy of the model, and the LSSVM parameters are optimized by the grid search method, i.e., the search range is set for the penalty factor *c* and the kernel function parameter *g* respectively, and the parameters are optimized within the specified interval, and the penalty factor *c* is finally determined to be 150 and the kernel function parameter *g* is 1.5. The parameters of the GM(1,1) model are set as follows: development The coefficient *a* is taken as 0.3, and the gray action quantity *b* is taken as 3.2.

**Figure 8:**
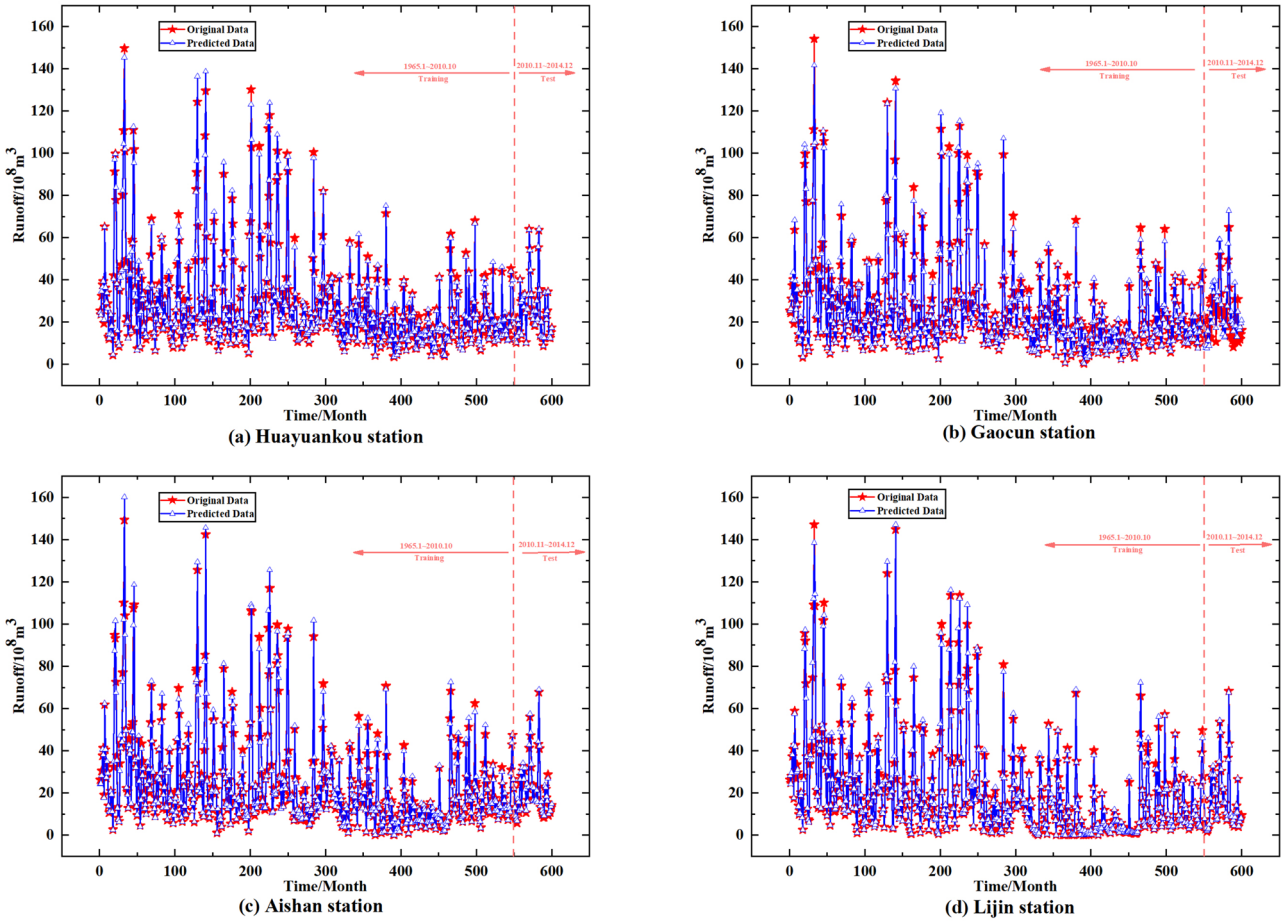
CEEMDAN–LSSVM–GM(1,1) model predicts monthly runoff results for stations under the Yellow River.

LSSVM was used to simulate the prediction of IMF1–IMF7 data from four hydrological stations obtained by CEEMDAN decomposition, and GM(1,1) was also used to simulate the prediction of Res obtained by CEEMDAN decomposition, and the prediction results were summed to obtain the monthly runoff prediction data of four hydrological stations, in which 550 months of data from January 1965 to October 2010 were The data from November 2010 to December 2014 were used for training and validation, and the monthly runoff predictions for the four hydrological stations in the lower Yellow River are shown in Fig. 8.

**Figure 9:**
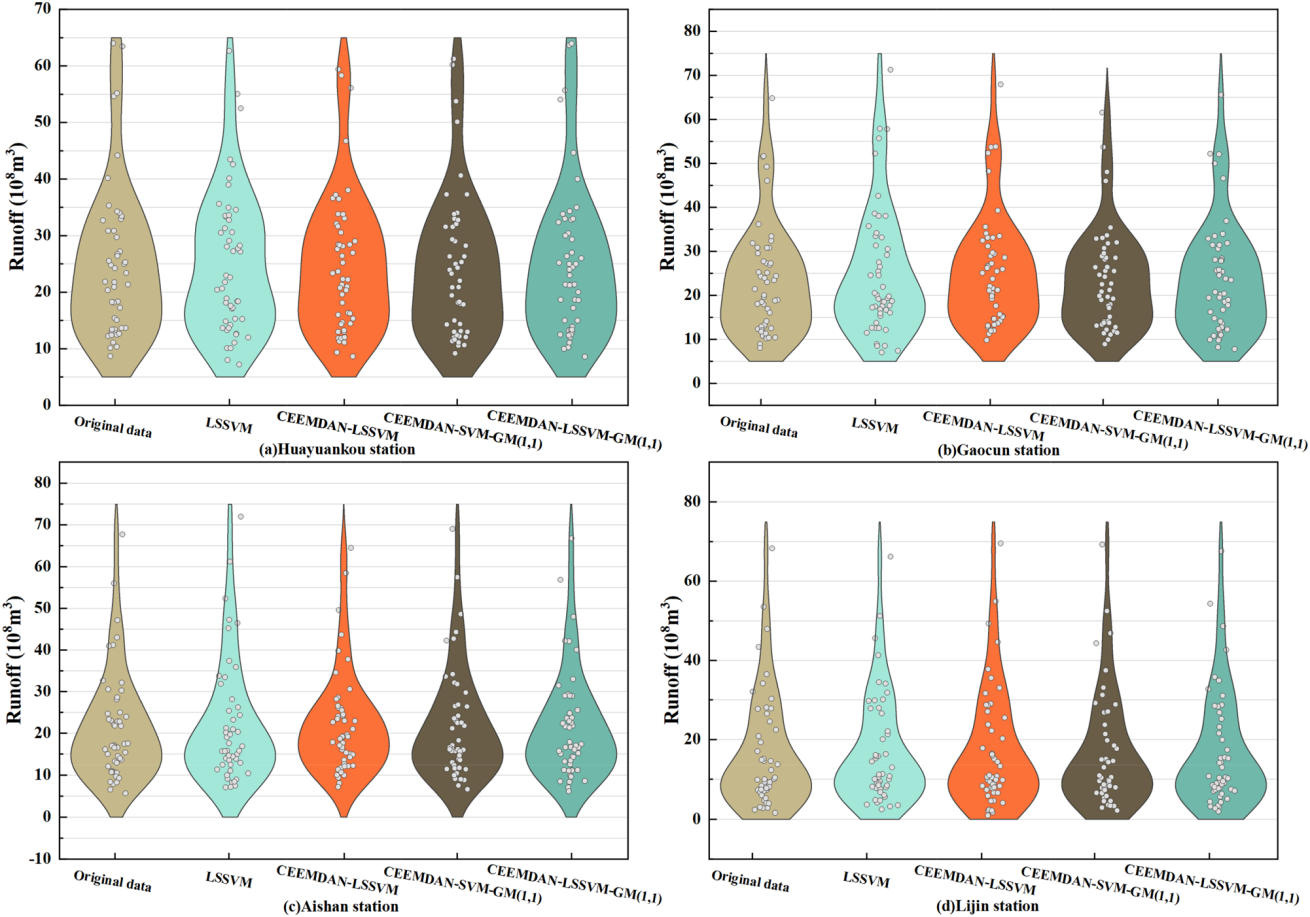
Comparison of the prediction results of the four models.

In order to verify the finiteness, accuracy and robustness of the CEEMDAN–LSSVM–GM(1,1) model for monthly runoff prediction, the prediction results of the LSSVM, CEEMDAN–LSSVM, CEEMDAN–SVM–GM(1,1) and CEEMDAN–LSSVM–GM(1,1) models were compared and analyzed in this paper, As shown in Fig. 9, the prediction accuracy of CEEMDAN–LSSVM–GM(1,1) model is the highest, the prediction accuracy of LSSVM model is poor, and the prediction results of the other two models are basically consistent with the original data.

### Discussion

The Nash–Sutcliffe efficiency coefficient (NSE), mean absolute error (MAE), mean absolute percentage error (MAPE), root mean squared error (RMSE) were calculated based on the prediction results of the four models. The results are shown in Table 1. The CEEMDAN–LSSVM–GM(1,1) model has the highest prediction accuracy, in which the NSE of Huayuankou station is 0.9521, MAE is $2.807 \times 10^{7} \;{\text{m}}^{3}$, MAPE is 1.20%, RMSE is $3.348 \times 10^{7} \;{\text{m}}^{3}$, NSE of Gaocun station is 0.9345, MAE is $5.434 \times 10^{7} \;{\text{m}}^{3}$, MAPE is 2.02%, RMSE is $5.638 \times 10^{7} \;{\text{m}}^{3}$, NSE of Aishan station is 0.9334, MAE is $6.221 \times 10^{7} \;{\text{m}}^{3}$, MAPE is 1.60%, RMSE is $4.874 \times 10^{7} \;{\text{m}}^{3}$, NSE of Lijin station is 0.9214, MAE is $7.442 \times 10^{7} \;{\text{m}}^{3}$, MAPE is 1.53%, RMSE is $4.687 \times 10^{7} \;{\text{m}}^{3}$. The Nash efficiency coefficients are all above 0.9, the mean absolute error $\le 7.442 \times 10^{7} \;{\text{m}}^{3}$ and the average absolute percentage error is around 2%. Zhang et al. (2020) used modified ensemble empirical mode decomposition (MEEMD)-autoregressive integrated moving average (ARIMA), to predict the runoff from 2010 to 2014 at Huayuankou hydrological station with a relative error of, and an average absolute percentage error of 6.04%^37^. Zhang et al.^38^ used the CEEMDAN-autoregressive moving average (ARMA) model to predict the runoff from 1960 to 2017 at Tang Naihai hydrological station in the Yellow River source area, and the prediction results showed a Nash efficiency coefficient of 0.786 and an average absolute percentage error of 8.78%, indicating that the CEEMDAN–LSSVM–GM(1,1) model has high prediction accuracy and good quality, and the credibility of the model is high.

**Table 1:** Evaluation of the prediction results of each model.

Station	Model	NSE	MAE/10^8^ m^3^	MAPE/%	RMSE/10^8^ m^3^
Huayuankou	LSSVM	0.8194	4.2318	17.63	5.6552
	CEEMDAN–LSSVM	0.8825	2.6074	10.67	3.2780
	CEEMDAN–SVM–GM(1,1)	0.9206	1.3158	5.40	1.6254
	CEEMDAN–LSSVM–GM(1,1)	0.9521	0.2807	1.20	0.3348
Gaocun	LSSVM	0.7936	4.5262	18.36	5.9261
	CEEMDAN–LSSVM	0.8554	2.0248	10.82	3.0628
	CEEMDAN–SVM–GM(1,1)	0.9026	1.0531	5.06	1.5228
	CEEMDAN–LSSVM–GM(1,1)	0.9345	0.5434	2.02	0.5638
Aishan	LSSVM	0.7832	4.3806	16.75	6.1321
	CEEMDAN–LSSVM	0.8467	2.5610	10.51	3.2416
	CEEMDAN–SVM–GM(1,1)	0.9143	1.2525	5.24	1.4852
	CEEMDAN–LSSVM–GM(1,1)	0.9334	0.6221	1.60	0.4874
Lijin	LSSVM	0.8382	5.0214	16.84	5.8113
	CEEMDAN–LSSVM	0.8847	3.2225	11.32	3.4562
	CEEMDAN–SVM–GM(1,1)	0.9016	1.3935	6.23	1.4895
	CEEMDAN–LSSVM–GM(1,1)	0.9214	0.7442	1.53	0.4687

**Figure 10:**
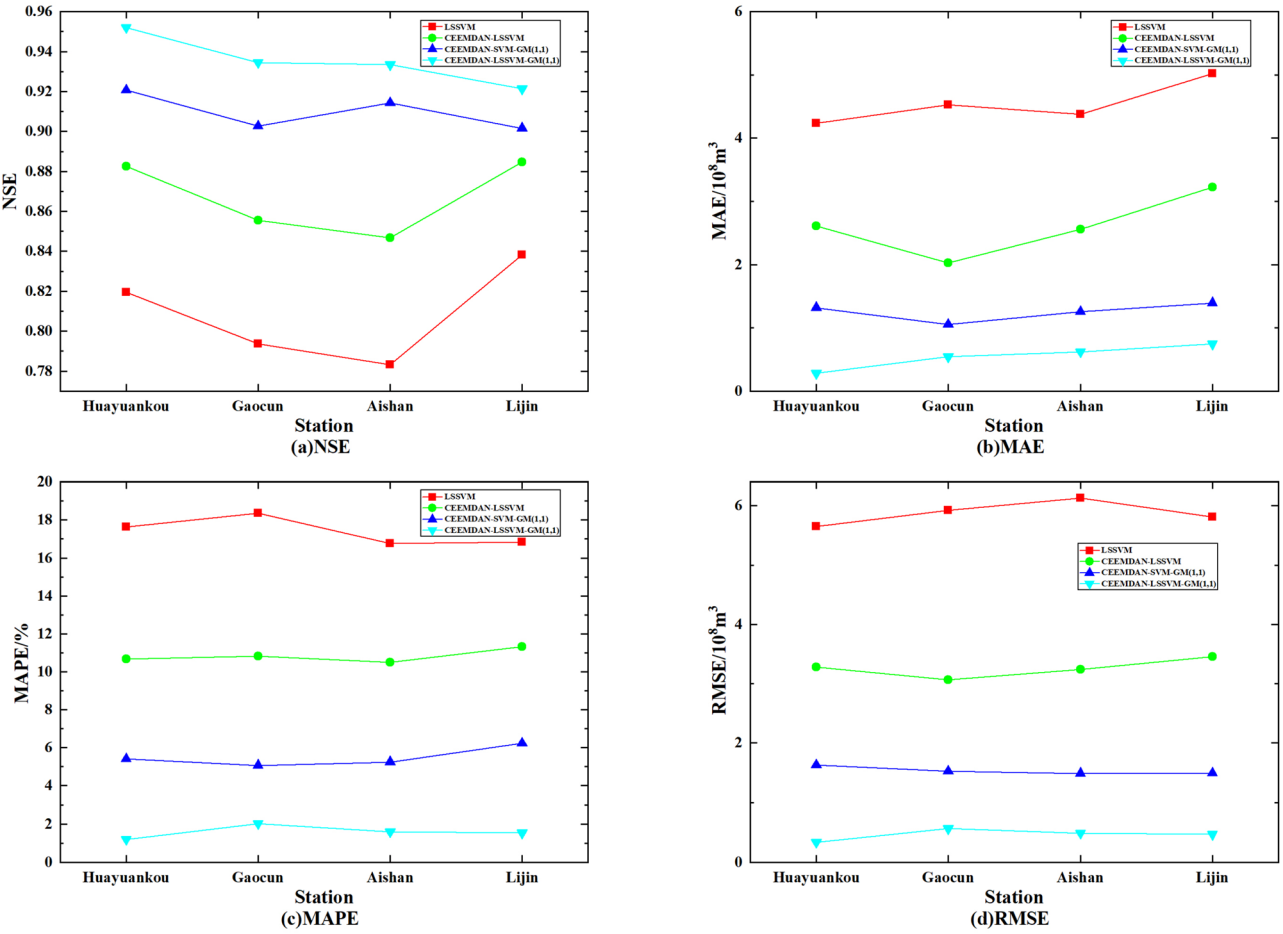
Comparison of four model evaluation indicators.

The evaluation metrics of the prediction results of the four models are shown in Fig. 10. Compared to the LSSVM model, the prediction results of the CEEMDAN–LSSVM–GM(1,1) model showed an improvement of 15.68% in NSE, 87.94% in MAE, 90.87% in MAPE and 92.12% in RMSE; compared to the CEEMDAN–LSSVM model, NSE improved by 7.84%, MAE reduced by 78.97%, MAPE reduced by 85.34% and RMSE reduced by 85.78%; compared to CEEMDAN–SVM–GM(1,1) model, NSE improved by 2.81%, MAE reduced by 56.32%, MAPE reduced by 71.04% and RMSE by 69.71%.

**Figure 11:**
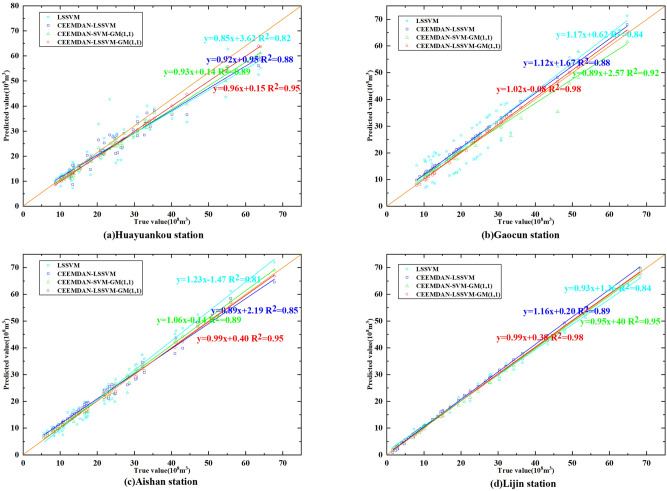
Scatter chart of four model prediction results.

Figure 11 shows the scatter plots of predicted versus observed values for each model at each station in the lower Yellow River and the corresponding linear trend lines for the scatter plots. The CEEMDAN–LSSVM–GM(1,1) model has a linear trend line closest to ${\text{y}} = x$ and therefore has optimal runoff simulation and prediction capabilities.

## Conclusion

1. The results of the monthly runoff prediction for four hydrological stations in the lower Yellow River show that the CEEMDAN–LSSVM–GM(1,1) model proposed in this paper has good accuracy and robustness. The highest prediction accuracy is at Huayuankou station, the highest Nash efficiency coefficient is at Huayuankou station with 0.9521, MAE is $2.807 \times 10^{7} \;{\text{m}}^{3}$, MAPE is 1.20%, RMSE is $3.348 \times 10^{7} \;{\text{m}}^{3}$, the lowest Nash efficiency coefficient is at Lijin station with 0.9214, the highest MAE is at Lijin station with $7.442 \times 10^{7} \;{\text{m}}^{3}$, MAPE The largest is Gaocun station with 2.02% and the largest RMSE is Gaocun station with $5.638 \times 10^{7} \;{\text{m}}^{3}$. Its prediction accuracy is higher than that of the LSSVM model, the CEEMDAN–LSSVM model, and the CEEMDAN–SVM–GM(1,1) model. This indicates that the CEEMDAN–LSSVM–GM(1,1) model is feasible for monthly runoff prediction and can be effectively used for time series analysis in hydrology and related fields to guide the rational development and improve the utilization of water resources.
2. The CEEMDAN–LSSVM–GM(1,1) model proposed in this paper can reduce prediction errors, improve data fitting ability and model stability to a large extent through data pre-processing-decomposition-noise reduction-prediction, and can be used as one of the means to enrich and improve the decomposition of medium and long-term runoff prediction.
3. Although the CEEMDAN–LSSVM–GM(1,1) model has a promising application with its effective decomposition algorithm and stable and fast prediction capability. Due to the problem of the model, the lag brought by physical mechanisms such as precipitation on runoff cannot be considered, and the input can only be runoff time series, which is a shortcoming of the model and a focus of further research in the future.

## Data Availability

Data and materials are available from the corresponding author upon request.
